# Nighttime dissolution in a temperate coastal ocean ecosystem increases under acidification

**DOI:** 10.1038/srep22984

**Published:** 2016-03-18

**Authors:** Lester Kwiatkowski, Brian Gaylord, Tessa Hill, Jessica Hosfelt, Kristy J. Kroeker, Yana Nebuchina, Aaron Ninokawa, Ann D. Russell, Emily B. Rivest, Marine Sesboüé, Ken Caldeira

**Affiliations:** 1 Department of Global Ecology, Carnegie Institution for Science, 260 Panama Street, Stanford, California, 94305, USA; 2 Bodega Marine Laboratory, University of California at Davis, Bodega Bay, California, 94923, USA; 3 Department of Earth & Planetary Sciences, University of California, Davis, CA 95616, USA; 4 Department of Ecology and Evolutionary Biology, Santa Cruz, California, 95064, USA

## Abstract

Anthropogenic emissions of carbon dioxide (CO_2_) are causing ocean acidification, lowering seawater aragonite (CaCO_3_) saturation state (Ω_arag_), with potentially substantial impacts on marine ecosystems over the 21^st^ Century. Calcifying organisms have exhibited reduced calcification under lower saturation state conditions in aquaria. However, the *in situ* sensitivity of calcifying ecosystems to future ocean acidification remains unknown. Here we assess the community level sensitivity of calcification to local CO_2_-induced acidification caused by natural respiration in an unperturbed, biodiverse, temperate intertidal ecosystem. We find that on hourly timescales nighttime community calcification is strongly influenced by Ω_arag_, with greater net calcium carbonate dissolution under more acidic conditions. Daytime calcification however, is not detectably affected by Ω_arag_. If the short-term sensitivity of community calcification to Ω_arag_ is representative of the long-term sensitivity to ocean acidification, nighttime dissolution in these intertidal ecosystems could more than double by 2050, with significant ecological and economic consequences.

The oceanic uptake of CO_2_ has increased due to anthropogenic CO_2_ emissions^1^. This process, often referred to as ‘ocean acidification’, has decreased global surface ocean pH by ~0.1 since the preindustrial era^2^ and is projected to further decrease pH by 0.07 to 0.33 units by 2100^3^. The ongoing reduction of the calcium carbonate saturation state of seawater, contemporaneous with ocean acidification^4^, is likely to affect the ability of many marine calcifiers to form their calcium carbonate shells or skeletons and is projected to have significant impacts on ocean ecosystems on decadal to millennial timescales^5^,^6^.

Calcification rates are a common indicator of individual or ecosystem health^7^. In laboratory manipulations, many calcifying species, including temperate macroalgae^6^,^8^,^9^ and invertebrates^10^,^11^,^12^, exhibit reduced rates of calcification in response to a reduction in the seawater saturation state. Consequently, *in situ* observations of the sensitivity of calcifying communities to natural saturation state variability are increasingly valued^13^, as they incorporate complex species interactions, and capture the carbonate chemistry conditions to which communities are acclimatised. Such analyses may therefore better represent the community level sensitivity to long-term ocean acidification. Studies have typically focused on sites with volcanic CO_2_ seeps that produce strong spatial gradients in calcium carbonate saturation state^13^,^14^. However, an alternative approach has been applied at sites that are isolated from the open ocean during low tides and can therefore experience large temporal (hourly) variability in calcium carbonate saturation state due to localised photosynthesis and respiration^15^,^16^. It is this approach that is utilised in this study to investigate the sensitivity of calcifiers to saturation state variability in temperate intertidal ecosystems. The main difference between the two methodologies is that the sensitivity of a community exposed to large short-term variability in carbonate saturation state may differ from that community’s sensitivity to ocean acidification which operates over much longer decadal to centennial timescales. As such, greater care is required when making long-term inferences from temporally isolated study sites.

Here, we assess the community level sensitivity of temperate tide pool calcification rates to variability in the calcium carbonate saturation state. Our intertidal study site at Bodega Marine Reserve in Northern California experiences extreme variation in calcium carbonate saturation state at low tide due to photosynthetic activity and respiration occurring after the time at which the pools become isolated from the open ocean. As photosynthetic activity is largely dependent on temperature and photosynthetically active radiation (PAR), which vary on a diurnal timescale, whereas tide pool isolation is predominantly a function of tidal phase, we were able to separate the influence of calcium carbonate saturation state on calcification from the influence of temperature and PAR. This system therefore provides a unique opportunity to characterise the *in situ* short-timescale sensitivity of tide pool community calcification rates to changes in saturation state.

## Results

### Tide pool community structure

**Figure 1:**
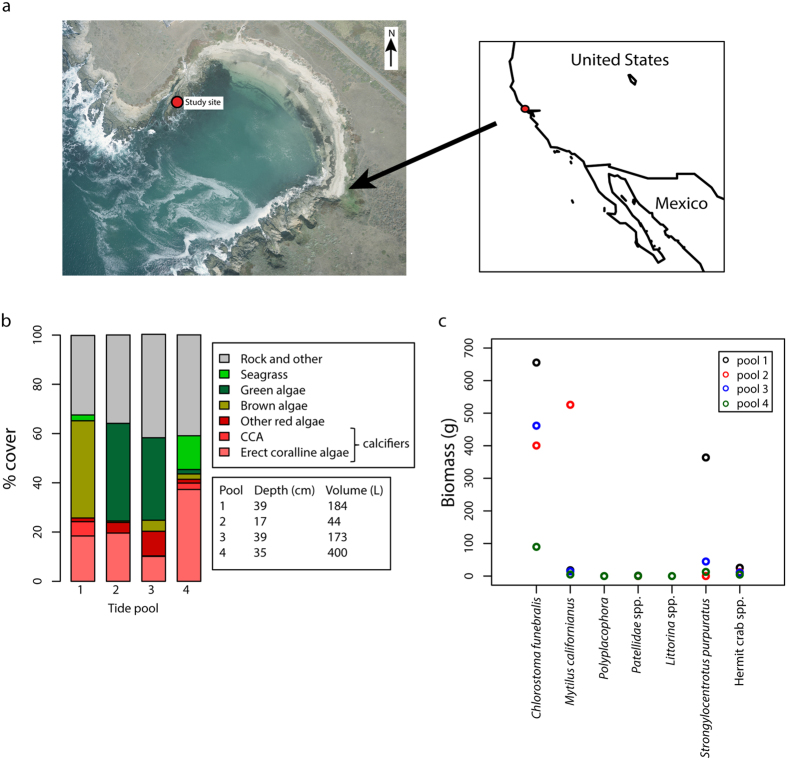
Study site characterisation. (**a**) An aerial photo of Horseshoe Cove, Bodega Marine Reserve, California and the location of the tide pool study site on the Northern California coast (38.3°N, 123.1°W), (**b**) the mean depth, volume and primary producer community cover and (**c**) the invertebrate community in each of the tide pools. The map is produced using R version 3.0.3 software (https://www.r-project.org/).

The mean depths, volumes, and community structure of the pools are given in Fig. 1. Mean pool depth varied from 17 cm to 39 cm while pool volume covered the range 44 L to 400 L. The dominant autotrophic calcifiers in the pools were coralline algae (e.g. *Corallina vancouveriensis* and *Calliarthron* sp.; 10.1–37.3% benthic cover) and crustose coralline algae (CCA; 0–5.8% benthic cover). The tide pool communities included various non-calcifying red algae (*Prionitis* sp. and *Mastocarpus* sp.; 1.5–10% benthic cover), brown algae (e.g. *Fucus vesiculosus*; 0.6–39.5% benthic cover), green algae (*Cladophora* sp. and *Enteromorpha* sp.; 0.04–39.6% benthic cover) and seagrasses (*Phyllospadix torreyi.*; 0.01–13.7% benthic cover). We note that pools also contained a diverse calcifying invertebrate community, including bivalves (i.e., *Mytilus californianus*) and gastropods (i.e., *Chlorostoma funebralis*, *Littorina* spp., *Polyplacophora* spp., and limpets). In the majority of pools, the dominant component of calcifying invertebrate biomass was *Chlorostoma funebralis* (Fig. 1c).

### Carbonate chemistry

**Figure 2:**
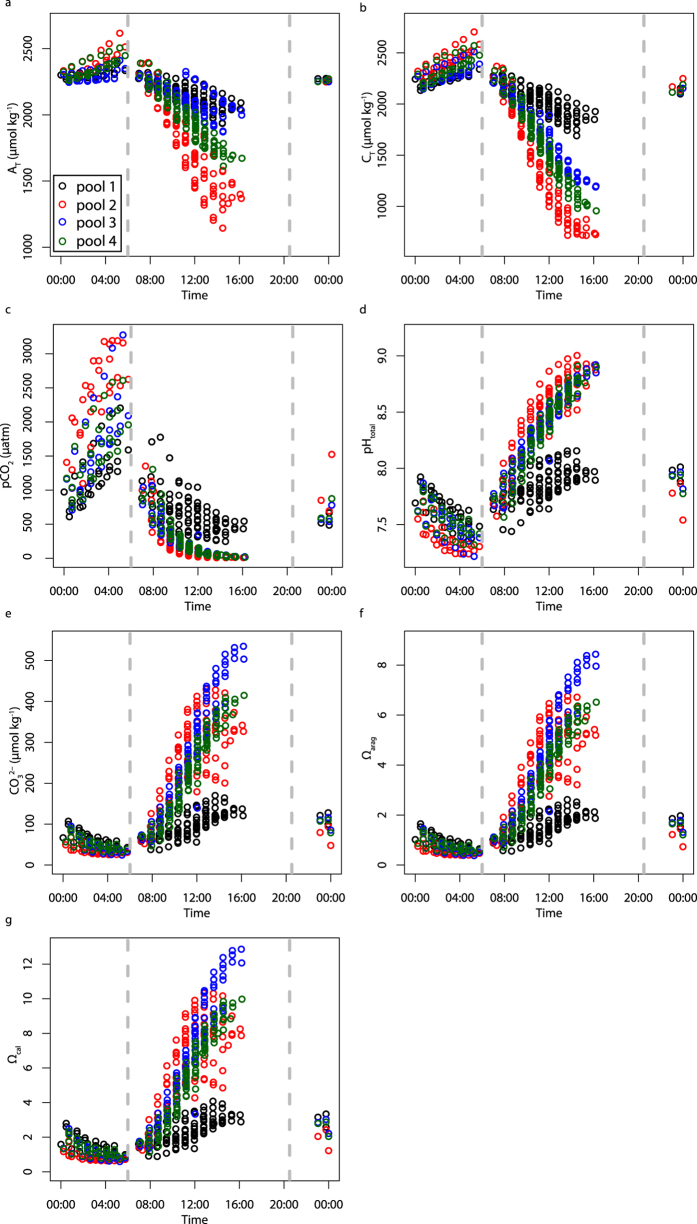
Carbonate chemistry parameters. (**a**) total alkalinity (*A*_*T*_; μmol kg^−1^), (**b**) dissolved inorganic carbon (*C*_*T*_; μmol kg^−1^), (**c**) *p*CO_2_ (μatm), (**d**) pH, (**e**) CO_3_^2−^ concentration (μmol kg^−1^), (**f**) aragonite saturation state (Ω_arag_) and (g) calcite saturation state (Ω_cal_) against time of day for all experimental time periods in each of the tide pools. Dashed grey lines show the approximate times of sunrise and sunset. Daytime data were collected in 2014 and nighttime data in 2015.

The carbonate chemistry of seawater in the tide pools varied substantially throughout the day in all pools studied (Fig. 2). Total alkalinity (*A*_*T*_) and dissolved inorganic carbon (*C*_*T*_) were highest before sunrise (maximum value of *A*_*T*_ = 2616 μmol kg^−1^; *C*_*T*_ = 2512 μmol kg^−1^) and decreased through the course of the day to minimum values in the late afternoon (minimum value of *A*_*T*_ = 1144 μmol kg^−1^; *C*_*T*_ = 715 μmol kg^−1^). The *p*CO_2_ showed similar declines, with a peak pre-sunrise value of 3276μatm and a minimum value of <10 μatm by mid-to-late afternoon. The pH, CO_3_^2−^ and Ω_arag_ all show large concurrent decreases through the night and increases during the day, with pH increasing from a minimum value before sunrise of 7.22 to a maximum late afternoon value of 9.00, and Ω_arag_ increasing through the day from a minimum value of 0.38 before sunrise to a maximum of 8.43. As discussed in greater detail below, the variation in carbonate chemistry parameters can be largely attributed to patterns of calcification, net primary production and changes in tide pool temperature.

While all four tide pools had broadly similar directional trends in carbonate chemistry parameters throughout the day, the magnitude of these trends shows large differences among pools. These differences are due to tide pool depth and the relative abundance of calcifying and photosynthesising taxa, in addition to the relative exposure of pools to incoming solar radiation. Pool 1, in particular, shows diel ranges in carbonate chemistry parameters of a much lower magnitude than the other pools (Fig. 2). Greater shading from incoming solar radiation and therefore lower pool temperature and PAR levels in Pool 1 may have enhanced the stability of its carbonate chemistry. The magnitude of carbonate chemistry changes in Pools 2, 3 and 4 seems to largely reflect pool depth, with shallower pools characterised by more substrate per pool volume (and thus greater benthic biomass per volume) exhibiting the largest daily reductions in *A*_*T*_ and *C*_*T*_ (Fig. 2).

### Daytime calcification

**Figure 3:**
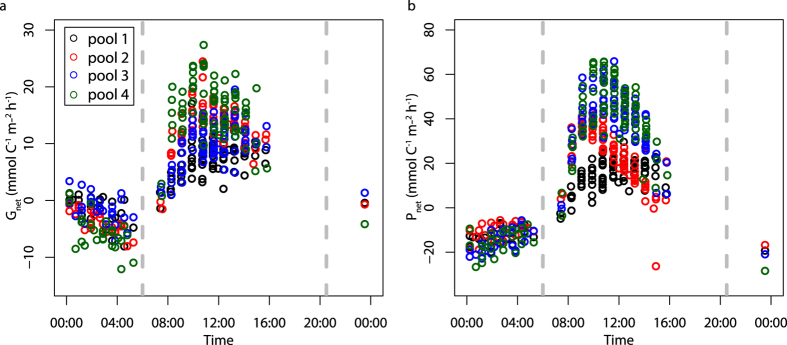
Temporal cycles in community calcification (G_net_) and production (P_net_). (**a**) G_net_ (mmol C^−1^ m^−2^ h^−1^) and (**b**) P_net_ (mmol C^−1^ m^−2^ h^−1^) against time of day in each of the tide pools. Dashed grey lines show the approximate times of sunrise and sunset. Daytime data were collected in 2014 and nighttime data in 2015.

Rates of daytime calcification are predominately positive, indicating net community calcification during daylight hours. Net community calcification (G_net_) shows similar diel trends to net community production (P_net_), with peak rates typically occurring between 11:00 and 13:00 (Fig. 3).

In all pools, G_net_ is linearly correlated with P_net_ (p < 0.05). G_net_ is linearly correlated to PAR, achieving statistical significance (p < 0.01) in all pools except for Pool 4 (p = 0.11). This is likely an artefact of the fewer measurements made at low light levels in Pool 4. While linearly correlated, the data suggest that the relationship between G_net_ and PAR can be better described by a Michaelis-Menten function with the positive influence of PAR on G_net_ saturating at PAR values >1000 μmol m^−2^ s^−1^ (Supplementary Fig. S4). G_net_ is linearly correlated to temperature (p < 0.05) and Ω_arag_ (p < 0.05) in all pools with the exception of Pool 4.

P_net_, PAR, and a Gaussian function of temperature (T_f_) explain 33–71% of the variation in daytime G_net_ in the tide pools studied (Table 1). Ω_arag_ offers no additional explanatory power in the prediction of daytime G_net_ for any of the tide pools, if variation in P_net_, PAR, and T_f_ is taken into account (Table 1).

### Nighttime calcification

**Figure 4:**
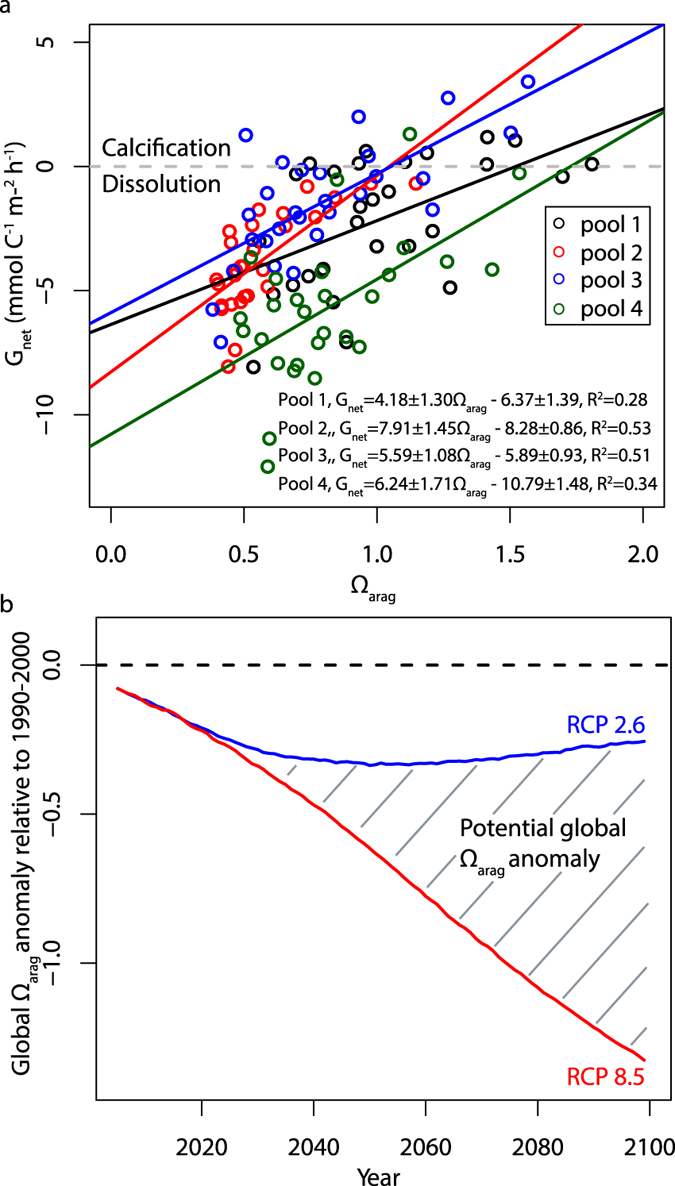
Nighttime community calcification and Ω_arag_ sensitivities/projections. (**a**) Nighttime G_net_ (mmol C^−1^ m^−2^ h^−1^) against aragonite saturation state (Ω_arag_) in each of the tide pools. Regression lines are significant at the p < 0.05 level. (**b**) Coupled Model Intercomparison Project Phase 5 (CMIP5) global Ω_arag_ anomalies relative to 1990–2000 mean values for the Representative Concentration Pathways 2.6 (RCP 2.6; blue) and 8.5 (RCP 8.5; red). Ω_arag_ anomalies are multimodel ensemble mean values calculated from 8 CMIP5 models with fully coupled ocean biogeochemistry schemes. RCP 2.6 and RCP 8.5 respectively represent the most extreme mitigation and business-as-usual RCP scenarios conducted in CMIP5.

Nighttime community calcification (G_net_) is found to be strongly influenced by Ω_arag_, with higher nighttime G_net_ at higher Ω_arag_ values in all tide pools. This is in contrast to the Ω_arag_ insensitivity described for daytime G_net_. Nighttime G_net_ typically occurs at lower Ω_arag_ values (0.38–2.06) than those present during the day (0.56–8.43), and is predominately negative, indicating net CaCO_3_ dissolution (Fig. 3). Ω_arag_ can explain 28–53% of the variability in nighttime calcification rates (Fig. 4a). Regression analyses indicate that the transition from net calcification to net dissolution occurs at Ω_arag_ values of 1.52, 1.05, 1.05 and 1.73 for Pools 1–4 respectively (Fig. 4a).

In the absence of other potential explanatory variables temperature was found to explain 20–45% of variability in nighttime G_net_ in 3 of the 4 pools (Fig. S6). P_net_ is found to only explain 21% of the variability in nighttime G_net_ in Pool 2 (Fig. S6) and none of the variability in nighttime G_net_ in the other tide pools. P_net_ is not present in the optimal nighttime model of Pool 2 (Table 1).

The dominant driver of nighttime G_net_ variability is Ω_arag_, with greater dissolution rates at lower values. Ω_arag_ is the only explanatory variable in the optimal model of calcification in each pool (Table 1) and explains 28 ± 9% of additional variability in nighttime G_net_ that is unaccounted for by P_net_ and T_f_. In total 47–62% of the variability in nighttime G_net_ can be explained by Ω_arag_, P_net_ and T_f_. Ω_arag_ has a coefficient of 6.16 ± 1.32, 7.91 ± 1.45 4.17 ± 1.11, and 8.70 ± 1.66 for Pools 1–4 respectively. As such, the Ω_arag_ sensitivity of nighttime calcification is broadly indistinguishable between tide pools although Pool 3 is less sensitive to Ω_arag_ variability than Pools 2 and 4.

## Discussion

The finding that Ω_arag_ offers no additional explanatory power in the prediction of daytime G_net_, if variation in P_net_, PAR, and temperature is taken into account (Table 1), suggests that at least in the short-term, daytime net calcification rates of intertidal communities are resilient to a very broad range of Ω_arag_ values (0.56–8.43). Daytime calcification rates could exhibit some dependency on Ω_arag_ as has been shown for individual species in laboratory manipulations that encompass a similar Ω_arag_ range^10^,^12^,^17^ but due to multicollinearity between Ω_arag_ and other potential explanatory variables such as PAR, this dependence may not be detectable *in situ*^18^,^19^. Such dependency however is highly unlikely given that the multicollinearity between explanatory variables is weak in our study (Supplementary Tables S1 and S2).

The positive correlations between G_net_ and P_net_ during the daytime imply that calcification and photosynthesis are intimately linked in these tide pools. This suggests that photosynthesis could enhance calcification processes during daylight hours^20^,^21^ and promote greater calcification resilience to variability in Ω_arag_. In the intertidal communities studied here, photosynthetic calcifiers (i.e., calcifying algae) may have a larger influence on carbonate chemistry dynamics than non-photosynthetic calcifying species, such as molluscs, during daylight hours. While we did not assess the extent to which individual taxa contribute to net community calcification in the tide pools, our results allow for predictions about the vulnerability of net daytime community calcification to variability in Ω_arag_ across a range of community compositions.

As shown in Fig. 4b, the decline of global mean Ω_arag_ as projected by an ensemble of current-generation Earth System Models, is highly dependent on the economic and environmental pathway taken. Under a business-as-usual scenario (RCP 8.5), global mean Ω_arag_ is projected to decline by 1.32 by the year 2100, relative to global mean Ω_arag_ in 1990–2000. Even under an extremely ambitious pathway aimed to limit global mean temperature rise to 2 °C (RCP 2.6), which assumes full participation in emission reductions by all countries, and the possibility of negative emissions^22^, Ω_arag_ is still expected to decline by up to 0.33 throughout the 21^st^ Century, relative to 1990–2000 mean Ω_arag_. Although the extent to which global declines in Ω_arag_ are reflected in intertidal zones will vary regionally and be mediated by local processes^23^, it is apparent that projected declines in Ω_arag_ are considerably smaller than our measured diel variability due to photosynthesis and respiration. The impact of ocean acidification in intertidal ecosystems will therefore be a consequence of the sensitivity of communities already exposed to a wide Ω_arag_ range experiencing a relatively smaller decline in mean background Ω_arag_^24^.

The statistical models derived for nighttime calcification all contain Ω_arag_, with P_net_ and a Gaussian function of temperature (T_f_) found to offer additional explanatory power in certain tide pools. Ω_arag_ coefficients in these models are between 4.17 ± 1.11 and 8.70 ± 1.66 depending on the tide pool. Therefore, a decline in background Ω_arag_ values of 0.5, which is highly likely by 2050 under even optimistic scenarios of future climate change (Fig. 4b)^4^,^25^, would reduce nighttime calcification (increase nighttime dissolution) by approximately 2.09–4.35 mmol C^−1^ m^−2^ h^−1^. Given that we measure mean nighttime calcification rates of −3.24 mmol C^−1^ m^−2^ h^−1^, this represents a decline in net community calcification of approximately 65–134% based on the statistical relationships derived in our experiment. It should be noted that our *in-situ* methodology does not distinguish between organism and sediment CaCO_3_ dissolution. Therefore additional controlled lab and mesocosm studies are required to isolate the biotic community sensitivity to Ω_arag_ variability and demonstrate that it does not significantly differ from the aggregate community sensitivity observed *in situ*.

Our estimates of the sensitivity of intertidal communities to Ω_arag_ responses recorded on hourly time scales only partially reflect the potential long-term response of ecosystems to decadal to millennial scale changes brought about by anthropogenic carbon dioxide emissions. Indeed, the sensitivity of intertidal organisms to Ω_arag_ in manipulative experiments has been shown to exhibit dependence on experimental duration^26^,^27^ and therefore the short-term Ω_arag_ sensitivity of the communities characterised here may differ from their long-term sensitivity to ocean acidification. Moreover, the overall impacts of ocean acidification and declining Ω_arag_ on temperate calcifiers will be more diverse and extend beyond the processes we evaluated, including potential impacts on survival and reproduction^7^. Nonetheless, if the short-term sensitivity of community calcification to Ω_arag_ conditions ranging from 0.38 to 8.43 described here is representative of the long-term sensitivity of these intertidal communities to ocean acidification, then one would expect daytime net calcification rates to be relatively resilient to future Ω_arag_ declines. Nighttime net calcification rates however, would be expected to decline, reducing the long-term community calcification of these ecosystems, with important ecological and economic ramifications.

## Materials and Methods

### Oceanographic and geochemical measurements

Our study site consisted of 4 rocky tide pools located in Horseshoe Cove, Bodega Marine Reserve, California (38.3°N, 123.1°W; Fig. 1). Over 5 sampling periods in April, May and June 2014, and May and June 2015, instruments were deployed in each of the tide pools over low tide periods (~7 hours, during which the pools were isolated from the open ocean). The sampling periods in 2014 occurred during daylight hours, while the sampling periods in 2015 occurred at night. The influence of interannual variability on comparisons between the 2014 and 2015 datasets is assumed to be minimal. This is supported by the agreement between 2014 and 2015 carbonate chemistry measurements in the crepuscular periods. Monthly differences between the PAR levels at a given time of day were minimal once periods with overcast conditions are taken account of. We utilised conductivity-temperature-depth sensors (CTDs; YSI model 6600 and 6920) to measure pressure, temperature, conductivity, pH, and oxygen at intervals of 2 minutes. When oxygen levels were high and interfered with conductivity measurements, salinity values were estimated from contemporaneous measurements in other tide pools. Water pumps attached to CTDs were used to take 1 L bottle samples every 50 minutes over the course of instrument deployment for discrete geochemical analyses (below). Seawater samples were analysed immediately for alkalinity and pH and preserved with HgCl_2_ for dissolved inorganic carbon (*C*_*T*_) analysis.

In total, 566 seawater samples were analysed for total alkalinity (*A*_*T*_), pH, dissolved inorganic carbon (*C*_*T*_ and nutrients (NO_2_^−^ + NO_3_^−^ and NH_3_ + NH_4_^+^). *A*_*T*_ was measured using a Metrohm 855 autotitrator. Three replicates of each sample were run, and the mean value of the two closest replicates was used (<2 μmol kg^−1^ (1SD) instrument precision). Titrant was standardised using reference material supplied by the Dickson Lab (Batch 141). The pH and O_2_ values measured via CTD were calibrated using spectrophotometric pH measurements and Winkler titrations, respectively, conducted according to best practices^28^ on discrete water samples. *C*_*T*_ was measured by infrared adsorption via a LICOR7000 CO_2_/H_2_O analyser coupled with a custom-made sample delivery system built by Stanford University’s Stable Isotope Laboratory (<2 μmol kg^−1^ (1SD) instrument precision). Nutrients were measured photometrically using a WestCo SmartChem 200 discrete analyser (~0.05 μmol L^−1^ instrument precision). The *p*CO_2_, CO_3_^2−^ and aragonite saturation state (Ω_arag_) values were calculated with *A*_*T*_ and *C*_*T*_ for 2014 data, and *A*_*T*_ and pH for 2015 data, using the CO2SYS program^29^. The K_1_ and K_2_ constants of Mehrbach *et al.*^30^ refit by Dickson and Millero^31^ were utilised, and total boron was calculated using the B/chlorinity ratio of Uppstrom 1974^32^.

Photosynthetically-active radiation (PAR) data were provided by the Bodega Ocean Observing Node, University of California, Bodega Marine Laboratory (http://bml.ucdavis.edu/boon/index.html). This record is measured within 100 m of the tide pools and is representative of local conditions. However, we note that local topography, water depth, and turbidity are additional influences on the direct PAR that tide pool communities received during the sample collection periods.

### Quantification of tide pool volume and community composition

Tide pool volumes were measured by draining each of the 4 tide pools and measuring the volume of drained water. The species composition and community structure of each pool was measured by overlaying a flexible mesh net with 10 × 10cm grid cells over the bottom of the emptied tide pool. The percent cover of all autotropic species and key functional groups was estimated on a 0 to 4 scale in each grid cell, with 0 denoting absence, 1 = 25%, 2 = 50%, 3 = 75%, and 4 = 100% cover for each taxa. The numerical values were then summed to estimate the total percent cover of each taxa within a tide pool. Invertebrate biomass values were estimated by weighing a representative sample of each taxa.

### Net community calcification and production

Net community calcification (G_net_; mmol C^−1^ m^−2^ h^−1^) was calculated in each tide pool using the salinity-normalised alkalinity anomaly method^28^:


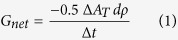


where Δ*A*_*T*_ is the change in salinity-normalised *A*_*T*_ (μmol kg^−1^), *d* is the mean tide pool depth (m), *ρ* is the density of sea water (kg m^−3^)^33^ and *Δt* is the time period (h). Net community production (photosynthesis minus respiration; P_net_; mmol C^−1^ m^−2^ h^−1^) is calculated as:


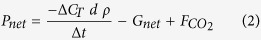


where Δ*C*_*T*_ is the change in salinity normalised *C*_*T*_ (μmol kg^−1^) and 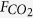 (μmol kg^−1^ m^−2^ h^−1^) is the air-sea flux of CO_2_ calculated using the wind speed-dependent gas transfer function of Ho *et al.* 2006^34^, the temperature and salinity dependent solubility of CO_2_ according to Weiss 1974^35^ and an atmospheric CO_2_ concentration of 401 ppm and 403 ppm for the 2014 and 2015 sampling periods respectively (the NOAA/ESRL Mauna Loa mean CO_2_ concentration over the sampling periods). Wind speed data were provided by the Bodega Ocean Observing Node, University of California, Bodega Marine Laboratory.

### Statistical modelling

Linear least square regression models were used to determine the influence of P_net_, PAR, temperature, and Ω_arag_ on the rate of community calcification (G_net_). Such an approach has been previously adopted in tropical environments (e.g. 16,36). Statistical issues involving multicollinearity between potential explanatory variables was assessed using Variance Inflation Factors (VIFs), which were all less than 6.5 (Supplementary Table S1). Model selection was based on minimising model AIC values with PAR-dependency assumed to follow a Michaelis-Menten function^20^ and temperature dependency assumed to follow a Gaussian function^37^ (Supplementary Fig. S4). The use of these functions as opposed to linear PAR and temperature relationships improved the explanatory power of models across tide pools. Thus, for each tide pool:


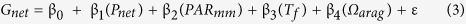


where Ω_arag_ is the mean of the omega aragonite values measured in two consecutive sampling times, PAR_mm_ is a Michaelis-Menten function of the mean PAR between two consecutive sampling times and T_f_ is a Gaussian function of the mean temperature between two consecutive sampling times. PAR_mm_ and T_f_ are derived separately for each tide pool from G_net_-PAR and G_net_-temperature relationships respectively (Supplementary Fig. S4). Note that equation 3 simplifies to the following for statistical models of nighttime calcification rates:


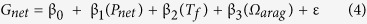


### Ω_arag_ projections

Coupled Model Intercomparison Project Phase 5 (CMIP5) global mean Ω_arag_ values are 2005–2100 annual anomalies calculated relative to the 1990–2000 mean values of the respective model. Values are GLobal Ocean Data Analysis Project (GLODAP)^38^, corrected and averaged across a multi-model ensemble of 8 CMIP5 Earth System Models which ran fully coupled ocean biogeochemistry schemes (CanESM2, GFDL-ESM2G, GFDL-ESM2M, IPSL-CM5A-LR, IPSL-CM5A-MR, MIROC-ESM, MPI-ESM-LR, MPI-ESM-MR). Ω_arag_ anomalies are given for the Representative Concentration Pathways 2.6 and 8.5^39^. RCP 2.6 and RCP 8.5 respectively represent the most extreme mitigation and business-as-usual RCP scenarios conducted in CMIP5 and are therefore considered to encompass the range of future potential Ω_arag_ values.

## Additional Information

**How to cite this article**: Kwiatkowski, L. *et al.* Nighttime dissolution in a temperate coastal ocean ecosystem increases under acidification. *Sci. Rep.* **6**, 22984; doi: 10.1038/srep22984 (2016).

## Supplementary Material

Supplementary Information

## Figures and Tables

**Table 1:** Statistical model parameter estimates for daytime and nighttime calcification.

Pool	Variable	Coefficient	Standard error	R^2^
**Daytime Calcification**				
1	PAR_mm_	25.74	4.00	0.34
2	P_net_	0.12	0.02	0.71
	PAR_mm_	8.62	1.60	
	T_f_	0.55	0.11	
3	PAR_mm_	25.43	4.16	0.33
4	P_net_	0.22	0.03	0.45
**Nighttime Calcification**				
1	Ω_arag_	6.16	1.32	0.47
	P_net_	0.37	0.12	
2	Ω_arag_	7.91	1.45	0.53
3	Ω_arag_	4.17	1.11	0.62
	T_f_	0.60	0.22	
4	Ω_arag_	8.70	1.66	0.53
	P_net_	0.26	0.08	

The optimal models of daytime and nighttime net calcification (G_net_) in each of the tide pools. During the night Ω_arag_ is the only potential explanatory variable in the optimal model of each tide pool. However during the day Ω_arag_ is found to offer no additional explanatory power in any of the tide pools. In each model all explanatory variables are significant at the p < 0.01 level.
