# Supplementary material for: Nighttime dissolution in a temperate coastal ocean ecosystem increases under acidification
Source: Sci Rep. 2016 Mar 18;6:22984. doi: 10.1038/srep22984 (PMC4796880; doi:10.1038/srep22984)
Supplement: Supplementary Information [file srep22984-s1.pdf]

Nighttime dissolution in a temperate coastal ocean ecosystem increases under acidification: Supplementary material

Lester Kwiatkowski[1]*, Brian Gaylord[2], Tessa Hill[2], Jessica Hosfelt[2], Kristy J. Kroeker[2,3], Yana Nebuchina[1], Aaron Ninokawa[2], Ann Russell[2], Emily B. Rivest[2], Marine Sesboüé[1], Ken Caldeira[1]

[1] Department of Global Ecology, Carnegie Institution for Science, 260 Panama Street, Stanford, California, 94305, USA;
[2] Bodega Marine Laboratory, University of California, Bodega Bay, California, 94923, USA;
[3] Center for Ocean Health, 100 Shaffer Road, University of California, Santa Cruz, California, 95064, USA.

Abbreviations

A_T	Total alkalinity
C_T	Dissolved Inorganic Carbon
G_{net}	Net community calcification
P_{net}	Net community production
Ω_{arag}	Aragonite saturation state
PAR	Photosynthetically active radiation
PAR_{mm}	Michaelis-Menten function of PAR
T_f	Gaussian function of temperature
F_{CO2}	Air-sea flux of CO_2
ρ	Sea water density
d	Mean tide pool depth

Collinearity between explanatory variables

Table S1. Variance Inflation factors (VIFs) for the potential explanatory variables in each tide pool during the day.

	PAR_{mm}	P_{net}	T_f	Ω_{arag}
**Pool 1**	5.48	2.00	4.32	1.28
**Pool 2**	2.49	1.22	1.64	2.15
**Pool 3**	6.45	2.25	5.04	4.00
**Pool 4**	3.07	2.69	2.11	2.26

Table S2. Variance Inflation factors (VIFs) for the potential explanatory variables in each tide pool during the night.

	P_{net}	T_f	Ω_{arag}
**Pool 1**	1.55	1.52	2.15
**Pool 2**	2.12	1.21	2.34
**Pool 3**	1.97	1.72	2.53
**Pool 4**	1.58	1.61	2.03

Table S3. Pearson correlation coefficients between explanatory variables in each tide pool during the day.

		PAR_{mm}	P_{net}	T_f	Ω_{arag}
**Pool 1**	PAR_{mm}	1			
	P_{net}	0.70	1		
	T_f	0.87	0.58	1	
	Ω_{arag}	0.43	0.29	0.46	1
**Pool 2**	PAR_{mm}	1			
	P_{net}	-0.09	1		
	T_f	0.58	0.18	1	
	Ω_{arag}	0.70	-0.29	0.37	1
**Pool 3**	PAR_{mm}	1			
	P_{net}	0.40	1		
	T_f	0.88	0.33	1	
	Ω_{arag}	0.71	-0.15	0.71	1
**Pool 4**	PAR_{mm}	1			
	P_{net}	0.53	1		
	T_f	0.37	0.71	1	
	Ω_{arag}	0.61	-0.03	-0.12	1

Table S4. Pearson correlation coefficients between explanatory variables in each tide pool during the night.

		P_{net}	T_f	Ω_{arag}
**Pool 1**	P_{net}	1		
	T_f	-0.09	1	
	Ω_{arag}	-0.55	0.53	1
**Pool 2**	P_{net}	1		
	T_f	-0.02	1	
	Ω_{arag}	-0.70	0.31	1
**Pool 3**	P_{net}	1		
	T_f	0.09	1	
	Ω_{arag}	-0.57	0.48	1
**Pool 4**	P_{net}	1		
	T_f	0.12	1	
	Ω_{arag}	-0.46	0.48	1

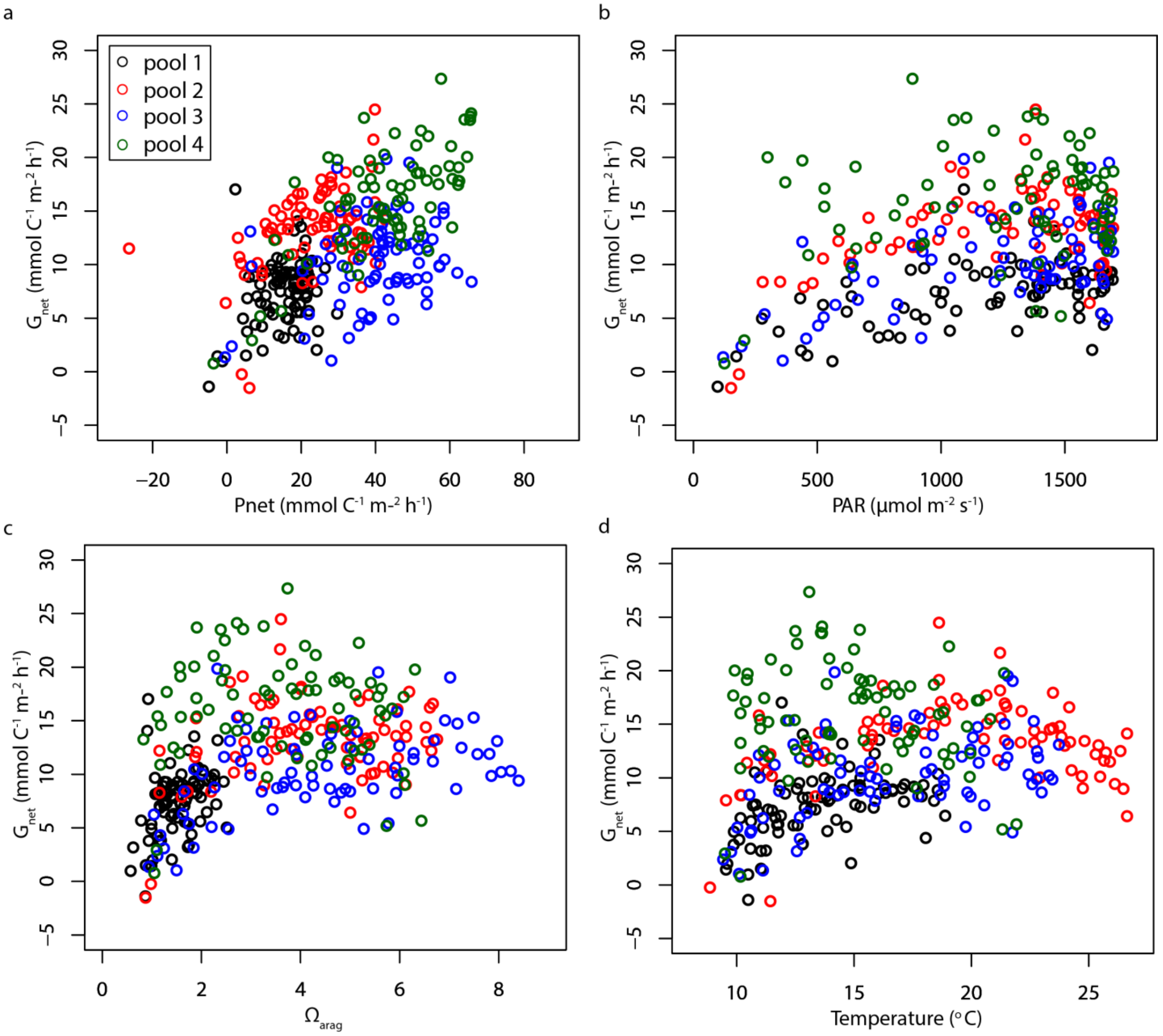

Figure S1. G_{net} (mmol C^{-1} m^{-2} h^{-1}) against a, P_{net} (mmol C^{-1} m^{-2} h^{-1}), b, Photosynthetically Active Radiation (µmol m^{-2} s^{-1}), c, Aragonite saturation state (Ω_{arag}) and d, Temperature (°C) in each of the tide pools during the day.

Nutrients

The concentration of NO_2^-+ NO_3^- is found to generally decrease between the first and last bottle samples on a given sample day (daylight hours; Fig. S2). Although this may be representative of net community nutrient NO_2^-+ NO_3^- uptake, the magnitude of changes are orders of magnitude lower than changes in ΔA_T and therefore any corresponding net community release of anions[1,2,3] would have negligible influence on G_{net}. The rate of change of NH_3 + NH_4^+ concentration between first and last daily bottle samples is shown to be highly variable (Fig. S2). However measured $\Delta(NH_3 + NH_4^+)$ explains none of the variance in ΔA_T (Fig. S3). As such, sporadic high concentrations are interpreted as a consequence of local animal excretion and not a result of net community uptake. The release of H^+ associated with the latter would have had a strong influence on measured A_T[1,2,3]. We therefore conclude that ΔA_T is primarily driven by community calcification. Nutrient concentrations were not measured for nighttime samples.

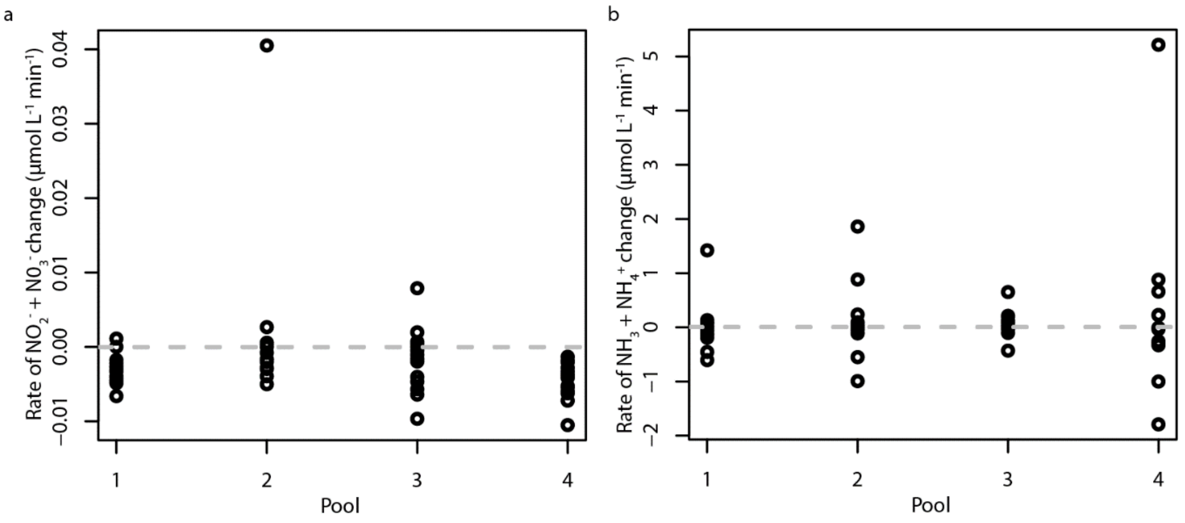

Figure S2. Rate of a, NO_2^- + NO_3^- and b, NH_3 + NH_4^+ concentration change between first and last daily sampling times in each tide pool (µmol L^{-1} min^{-1}). All samples were taken during the daytime.

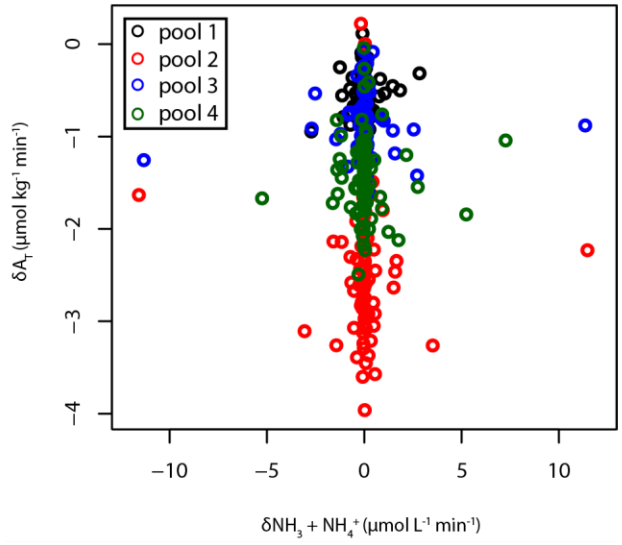

Figure S3. δNH_3 + NH_4^+ (µmol L^{-1} min^{-1}) against δA_T (µmol kg^{-1} min^{-1}) in each tide pool. There is no significant relationship between δNH_3 + NH_4^+ and δA_T in any of the tide pools.

PAR and temperature functions

Pool	Michaelis-Menten coefficients K_m	V_{max}
1	1019.7±487.0	14.16±3.00
2	402.7±123.9	18.23±1.44
3	815.3±361.2	17.83±3.08
4	147.4±71.9	18.29±1.18

a b

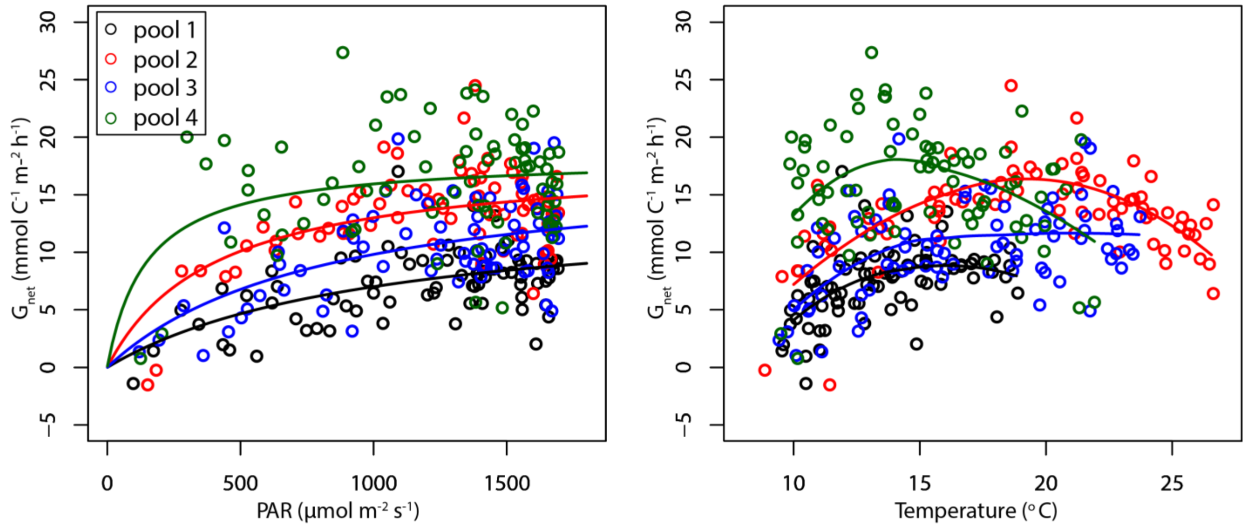

Figure S4. G_{net} (mmol C^{-1} m^{-2} h^{-1}) against a, PAR (µmol m^{-2} s^{-1}) and b, temperature (°C) in each of the tide pools. Michaelis-Menten and Gaussian functions are derived for G_{net}-PAR and G_{net}-temperature relationships respectively. Michaelis-Menten ($V=V_{max}[S]/(K_m+ [S])$) coefficients with associated standard errors are given however as the Gaussian temperature functions are produced using locally weighted regression methods (LOESS)[4] they cannot be represented by a simple mathematical equation.

Alkalinity-dissolved inorganic carbon relationships

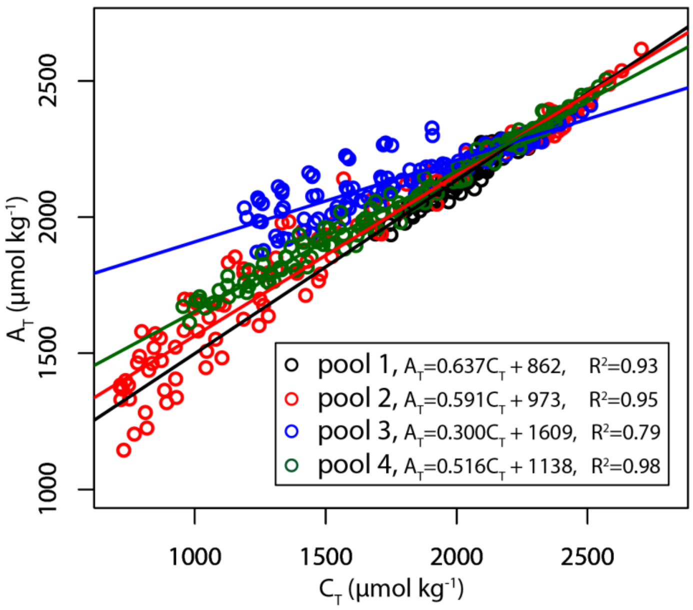

Figure S5. Total least squares regressions between alkalinity (A_T) and dissolved inorganic carbon (C_T) in each of the tide pools.

Nighttime community calcification against P_{net} and temperature

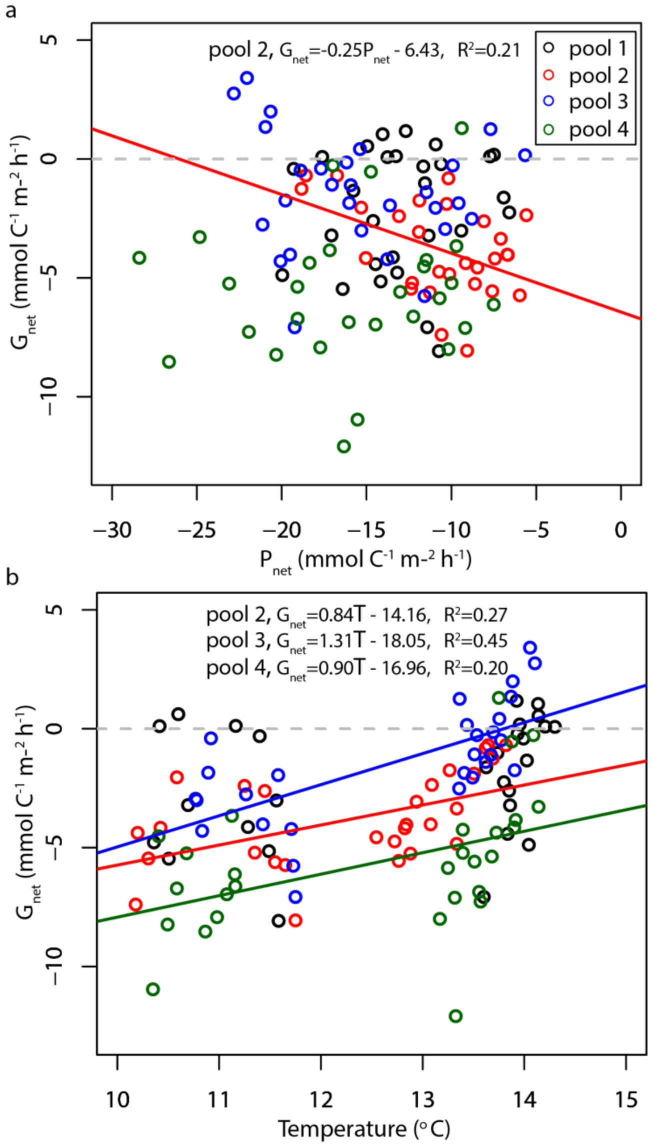

Figure S6. Nighttime G_{net} (mmol C^{-1} m^{-2} h^{-1}) against a, P_{net} (mmol C^{-1} m^{-2} h^{-1}), and b, Temperature (°C) in each of the tide pools. Regression lines of variables significant at the $p<0.05$ level are shown. Dashed grey lines show the separation between net community calcification and net community dissolution.

References

1. Brewer, P. G. & Goldman, J. C. Alkalinity changes generated by phytoplankton growth1. *Limnol. Oceanogr.* **21,** 108–117 (1976).
2. Goldman, J. C. & Brewer, P. G. Effect of nitrogen source and growth rate on phytoplankton-mediated changes in alkalinity1. *Limnol. Oceanogr.* **25,** 352–357 (1980).
3. Wolf-Gladrow, D. A., Zeebe, R. E., Klaas, C., Körtzinger, A. & Dickson, A. G. Total alkalinity: The explicit conservative expression and its application to biogeochemical processes. *Mar. Chem.* **106,** 287–300 (2007).
4. Cleveland, W. S. & Loader, C. in *Statistical Theory and Computational Aspects of Smoothing* (eds. Härdle, P. D. W. & Schimek, P. D. D. M. G.) 10–49 (Physica-Verlag HD, 1996). at <http://link.springer.com/chapter/10.1007/978-3-642-48425-4_2>
